# Personality impacts fear of childbirth and subjective birth experiences: A prospective-longitudinal study

**DOI:** 10.1371/journal.pone.0258696

**Published:** 2021-11-03

**Authors:** Eva Asselmann, Susan Garthus-Niegel, Julia Martini

**Affiliations:** 1 Differential and Personality Psychology, Faculty of Health, HMU Health and Medical University Potsdam, Potsdam, Germany; 2 Department of Psychology, Faculty of Life Sciences, Humboldt-Universität zu Berlin, Berlin, Germany; 3 Institute for Systems Medicine (ISM), Faculty of Human Medicine, MSH Medical School Hamburg, Hamburg, Germany; 4 Institute and Policlinic of Occupational and Social Medicine, Faculty of Medicine, Technische Universität Dresden, Dresden, Germany; 5 Department of Child Health and Development, Norwegian Institute of Public Health, Oslo, Norway; 6 Institute of Clinical Psychology and Psychotherapy, Faculty of Psychology, School of Science, Technische Universität Dresden, Dresden, Germany; 7 Department of Psychiatry and Psychotherapy, Faculty of Medicine, Carl Gustav Carus University Hospital, Technische Universität Dresden, Dresden, Germany; Kobe University Graduate School of Medicine School of Medicine, JAPAN

## Abstract

**Background:**

Previous research suggests that less emotionally stable, less conscientious, less extraverted, and less agreeable women tend to suffer from higher fear of childbirth and experience their delivery as worse. Moreover, there is evidence that birth characteristics and unexpected incidents during delivery may impact women’s birth experiences. However, it remains unknown whether the role of personality in subjective birth experiences varies between women with different birth characteristics.

**Methods:**

We used data from the Maternal Anxiety in Relation to Infant Development (MARI) Study, a regional-epidemiological study among pregnant women, who were prospectively followed up in multiple waves across the peripartum period. During pregnancy, personality was assessed with the short version of the Big Five Inventory. The Wijma Delivery Expectancy/ Experience Questionnaire was used to measure fear of childbirth (version A) during pregnancy and subjective birth experiences (version B) within the first 10 days after delivery.

**Results:**

Linear regressions revealed that lower levels of emotional stability, agreeableness, and extraversion predicted higher fear of childbirth during pregnancy. Moreover, personality affected subjective birth experiences especially in women with specific birth characteristics: Lower emotional stability predicted worse subjective birth experiences in women with (vs. without) a preterm delivery, and higher conscientiousness predicted worse subjective birth experiences in women with an emergency cesarean section (vs. spontaneous delivery). Subjective birth experiences were also worse in less emotionally stable and less open women with (general) anesthesia (vs. no anesthesia) during delivery. Finally, higher emotional stability predicted a subjective birth experience that was worse than expected, particularly in multiparous women and women without anesthesia during delivery.

**Conclusions:**

These findings suggest that less emotionally stable, less conscientious, and less open women tend to experience their delivery as worse particularly in case of unexpected incidents (i.e., preterm delivery, emergency cesarean section, and necessity of anesthetics) and might thus profit from early targeted interventions.

## Introduction

Giving birth to a child belongs to the most striking experiences in life. However, many pregnant women suffer from severe fear of childbirth (FOC) and experience their delivery in very different ways, depending on their personality, previous life and birth experiences, the pregnancy, and the circumstances of birth [1–7]. Women with severe FOC often avoid becoming pregnant, sometimes even terminate their pregnancy, or request a medically unnecessary cesarean section [8–10]. Anxiety during pregnancy, including severe FOC, may not only cause distress and burden to expectant mothers but also increase the risk of medical complications during pregnancy, labor, and delivery [1, 2, 11]. Consistently, higher FOC has been associated with unfavorable birth outcomes and worse subjective birth experiences [2].

Women who experience their delivery as particularly adverse tend to have greater difficulties to recover from their pregnancy and delivery (physically and mentally) and to adjust to the new family situation [12, 13]. Highly adverse subjective birth experiences have been associated with postpartum mental health problems (e.g., post-traumatic stress, anxiety, and depressive symptoms and disorders) [13–17]. These problems may not only impair women’s subjective well-being but also their social relationships (e.g., to their infant [18] and partner [16]) and increase the risk of developmental problems in the offspring [19–21]. Moreover, highly adverse birth experiences have been linked to sexual problems [22], a delay or avoidance of subsequent pregnancies [23], and severe FOC during subsequent pregnancies [24].

Therefore, risk factors for FOC and adverse subjective birth experiences need to be identified. Such research helps to recognize high-risk women as early as possible to prevent a vicious cycle of severe FOC, highly adverse subjective birth experiences, and associated complications [25–27]. The current study examines how personality relates to FOC and subjective birth experiences.

### The role of personality in FOC and subjective birth experiences

Personality refers to individual differences in thoughts, feelings, and behavior [28]. A large proportion of these individual differences can be captured with the Big Five personality traits openness to experience, conscientiousness, extraversion, agreeableness, and emotional stability [29].

Previous research found that especially less emotionally stable [30–36] but also less conscientious [30, 31, 33, 34, 37], less extraverted [30, 32, 34, 35], and less agreeable [30, 31] women reported higher FOC and worse subjective birth experiences. Emotionally stable people tend to be relatively resistant against stress and to experience less negative affect [38]. Thus, more emotionally stable women might worry less about their upcoming delivery [30–33, 35] and also experience their delivery as less adverse [34, 36].

Conscientious individuals tend to be organized, reliable, and perseverant and are typically characterized by a high sense of mastery and self-control [38, 39]. Therefore, more conscientious women might prepare their delivery more carefully (e.g., through doctoral/ hospital visits and birthing classes), be more confident to successfully manage their delivery, and thus experience lower FOC [30, 31, 33]. During delivery, more conscientious women might particularly profit from high levels of endurance and self-efficacy, which might favor the birth process. As a result, more conscientious women might be able to better cope with the situation and experience their delivery as less adverse [34, 37].

Extraverted people tend to be outgoing, socially active, and prone to experience positive emotions [40–42]. Therefore, more extraverted women might be more optimistic and experience their delivery as less adverse [30, 32, 34, 35]. Moreover, more extraverted women might have a larger social network and be more likely to share their birth-related fears with others, which could have stress-buffering effects [13, 43].

Agreeable individuals typically aim to be kind, friendly, cooperative, and trustful. Thus, more agreeable women might have more intimate and stable relationships (e.g., to their partner) and receive higher social support during pregnancy and delivery, which might lead to lower fear during pregnancy and less distress during delivery [13].

Findings concerning the role of openness in FOC and subjective birth experiences were less consistent [30, 31, 36]. Previous research either found that openness was unrelated to FOC [32, 33, 35] or related to lower [30] or higher [31] FOC. In one study, higher openness was associated with higher labor pain, a stronger reaction to labor pain, lower pain tolerance, and lower pain acceptance [36]. Openness relates to intelligence, creativity, imagination, and sensitivity [44, 45]. Open people tend to be open-minded and search for new ideas, activities, and experiences. However, this typically relates to intellectually stimulating mental experiences instead of painful physical experiences, which could explain inconsistent results [36].

Taken together, previous research suggests that especially lower emotional stability, but also lower conscientiousness, extraversion, and agreeableness relate to higher FOC and worse subjective birth experiences. However, whether the role of personality in subjective birth experiences varies by birth characteristics remains unresolved [37].

### The role of birth characteristics

#### Parity

Due to a lack of previous childbirth experiences, primiparous women might be more likely to experience their delivery as threatening and adverse. Therefore, personality might play a more important role for subjective birth experiences in primiparous vs. multiparous women. For example, especially first-time mothers with low levels of emotional stability might experience their delivery as more adverse.

#### Preterm delivery

As indicted by previous research, subjective birth experiences tend to be worse in women with (vs. without) a preterm delivery [46]. A preterm delivery often sets in unexpectedly and relates to an increased risk of unfavorable outcomes. Especially under such circumstances, personality might affect how women think, feel, and behave. In other words, personality might play a more important role for subjective birth experiences in women with (vs. without) a preterm delivery.

#### Mode of delivery and anesthesia

Previous research suggests that women with an instrumental vaginal delivery or an emergency cesarean section (which is typically conducted under general anesthesia) tend to experience their delivery as worse than women with a planned spontaneous delivery [47]. However, whether the role of personality in subjective birth experiences varies by mode of delivery and/ or use of anesthetics remains unresolved. On the one hand, personality might play a more important role under extraordinary circumstances (e.g., when an emergency cesarean section is necessary). On the other hand, it is plausible to assume that nearly every woman would experience such a “strong” situation as highly adverse (i.e., regardless of her personality). Therefore, one could also speculate whether personality plays a more important role for subjective birth experiences in women with a spontaneous delivery (vs. instrumental vaginal delivery or cesarean section) and in women without (vs. with) anesthesia.

### Aims of the study

Using prospective-longitudinal data from a regional-epidemiological sample of expectant mothers, this study aims to investigate the role of the Big Five personality traits in FOC and subjective birth experiences, considering the discrepancy between subjective birth experiences and previous FOC. Moreover, we examine whether the role of personality in subjective birth experiences varies by birth characteristics.

Our hypotheses are as follows: Lower emotional stability, lower conscientiousness, lower extraversion, and lower agreeableness relate to higher FOC and worse subjective birth experiences. The role of these personality traits in subjective birth experiences is greater in primiparous (vs. multiparous) women, in women with (vs. without) a preterm delivery, in women with a spontaneous delivery (vs. instrumental vaginal delivery or cesarean section), and in women without (vs. with) anesthesia during delivery.

## Materials and methods

### Procedure

The prospective-longitudinal Maternal Anxiety in Relation to Infant Development (MARI) Study was conducted in 306 expectant mothers, sampled from gynecological outpatient settings in the area of Dresden, Germany (study period: 01/ 2009 until 09/ 2012). These (expectant) mothers completed up to seven assessments: T1 (baseline): 10 to 12 weeks of gestation; T2: week 22 to 24 of gestation; T3: week 35 to 37 of gestation; T4: 10 days postpartum; T5: 2 months postpartum; T6: 4 months postpartum; and T7: 16 months postpartum.

Participants were investigated with standardized diagnostic interviews, questionnaires, and observations. Written informed consent was obtained from all participants. More detailed information on the objectives, methods, design, and inclusion/ exclusion criteria, including a detailed study flow chart, has been previously published [48, 49].

The study received approval from the Ethics Committee of the Medical Faculty of the Technische Universität Dresden (No: EK 94042007) and was carried out in accordance with the Helsinki Declaration of 1975, as revised in 2013. After consulting the Ethics Committee and due to the sensitive nature of the questions asked in this study, participants were assured that all raw data would remain confidential and would not be shared. Therefore, no openly assessable data files are attached. Further information on the data can be obtained from the corresponding author (Julia Martini, email: julia-martini@tu-dresden.de), the Ethics Committee of the Medical Faculty of the Technische Universität Dresden (email: ethikkommission@mailbox.tu-dresden.de), and the Institute of Clinical Psychology and Psychotherapy of the Technische Universität Dresden.

### Sample

In total, 533 pregnant women were screened for inclusion and exclusion criteria in gynecological outpatient settings in the area of Dresden (Germany). Fifty women were not enrolled due to specific exclusion criteria (gestational age > 12 weeks: *N* = 8; younger than 18 or older than 40 years: *N* = 8; multiple pregnancy: *N* = 2; history of more than three spontaneous abortions, (induced) termination of the pregnancy, still birth, or infant impairment: *N* = 2; invasive fertility treatment: *N* = 9; severe physical disease, microsomia, or skeletal malformation: *N* = 6; substance abuse or heroin substitution during the past six months: *N* = 0; severe psychiatric illness: *N* = 2; intention to leave the area of Dresden: *N* = 6; insufficient mastery of the German language: *N* = 7). In addition, 9 women did not participate due to a spontaneous abortion before T1, *N* = 10 because their partner did not agree, *N* = 154 due to a lack of time, and *N* = 4 due to unknown reasons.

Finally, 306 women participated at T1, 293 at T2, 278 at T3, 284 at T4, 281 at T5, 283 at T6, and 267 at T7. Due to a spontaneous abortion or termination of the pregnancy, the participation of 8 women ended after T1. During the study, 3 women moved away, 5 women could not be reached anymore by phone, postal, or personal contact, 9 women reported a lack of time or interest in a further participation, and 7 women refused to be contacted again for further follow-up assessments. Some retained women did not participate in single assessments, for example, due to a preterm delivery, sickness, or a lack of time (T3: *N* = 10; T4: *N* = 2; T5: *N* = 5; T6: *N* = 1; T7: *N* = 7). Detailed information on sociodemographic, gynecological, and clinical characteristics of the study sample, including information on systematic drop out from T1 to T7, has been previously presented [48, 49].

### Assessment of personality

The Big Five personality traits openness to experience, conscientiousness, extraversion, agreeableness, and emotional stability were assessed at T2 using the German short version of the Big Five Inventory (BFI-K) [50]. The BFI-K contains 21 items (5 items for openness and 4 items for each of the other traits), labeled from 1 = ‘strongly disagree’ to 5 = ‘strongly agree’. The reliability of the BFI-K has been shown to be acceptable, and the factorial validity and convergence of self-reports with partner ratings and other inventories have been supported [50].

### Assessment of FOC and subjective birth experiences

The Wijma Delivery Expectancy/ Experience Questionnaire (W-DEQ) [51] was used to measure FOC (version A) at T3 and subjective birth experiences (version B) at T4. The W-DEQ is the most widely used assessment instrument for FOC (version A) and subjective birth experiences (version B), operationalized as a uni-dimensional construct that captures women’s cognitive and emotional appraisal of the upcoming or past delivery. Women are asked to anticipate how the labor and delivery will be (W-DEQ-A) or how it was (W-DEQ-B) and how they will feel (W-DEQ-A) or have felt (W-DEQ-B). Both versions contain 33 items labeled from 1 = ‘not at all’ to 5 = ‘extremely’. Positively formulated items are reversed. The sum score of each version ranges from 0 to 165, with higher scores indicating higher FOC (W-DEQ-A) and worse subjective birth experiences (W-DEQ-B). Psychometric properties of the W-DEQ, including internal consistency and split-half-reliability, have been shown to be excellent [51–53].

### Assessment of sociodemographic and birth characteristics

Sociodemographic information (i.e., age, education, marital status, working time, and household income) was assessed with the Composite International Diagnostic Interview for Women (CIDI-V) [54]. Information on birth characteristics (i.e., parity, preterm delivery, mode of delivery, and anesthesia) was retrieved from medical records [55].

### Statistical analyses

Stata 14 [56] was used for the analyses. All women who provided information on FOC (W-DEQ-A, T3, *N* = 276) and/ or subjective birth experiences (W-DEQ-B, T4, *N* = 282) were considered (*N* = 286). Specifically, 272 women provided information on the W-DEQ-A and W-DEQ-B, 4 women provided information on the W-DEQ-A but not W-DEQ-B, and 10 women provided information on the W-DEQ-B but not W-DEQ-A.

First, we investigated the role of personality in FOC and subjective birth experiences. Specifically, we regressed (a) the W-DEQ-A score, (b) the W-DEQ-B score, and (c) the difference of both scores (W-DEQ-B minus W-DEQ-A score) on the Big Five personality traits. In crude models, only one trait was considered at a time. In multiple models, all five traits were considered simultaneously as multiple predictors.

Second, we examined the role of birth characteristics in subjective birth experiences, including the difference score between subjective birth experiences and previous FOC. Specifically, we regressed the respective outcome (i.e., W-DEQ-B score or W-DEQ-B minus W-DEQ-A score) on the respective birth characteristic.

Third, we tested whether the role of personality in subjective birth experiences, including the discrepancy between subjective birth experiences and previous FOC, varied by birth characteristics. Specifically, we built an interaction term of the respective Big Five trait * birth characteristic and then regressed the respective outcome (i.e., W-DEQ-B score or W-DEQ-B minus W-DEQ-A score) on this term. To avoid multicollinearity, one interaction was tested at a time.

All dimensional scores (i.e., the Big Five and W-DEQ scores) were standardized for the analyses (*M* = 0, *SD* = 1). The analyses were adjusted for age at baseline (T1). The alpha level was set at.05. No alpha adjustment was made because each analysis refers to a separate hypothesis [57].

## Results

### Sample characteristics

Sample characteristics, including means and standard deviations for FOC and subjective birth experiences, are presented in Table 1. On average, the sample reported moderate FOC (W-DEQ-A score: *M* = 84.05, *SD* = 9.23) and birth experiences that were moderately adverse (W-DEQ-B score: *M* = 88.23, *SD* = 10.71) [58] but worse than expected (W-DEQ-B minus W-DEQ-A score: *M* = 4.13, *SD* = 9.92). The correlation of both scores (i.e., W-DEQ-A and W-DEQ-B) was *r* = .49, and higher FOC predicted worse subjective birth experiences (*β* = 0.45, 95% CI: 0.35; 0.56, *p* < .001).

**Table 1:** Sample and birth characteristics of the current sample (N = 286)^1^.

**Sample characteristic**	**N**	**%**
**Age at T1 (M, SD)**	28.13	4.40
**Education**		
** No degree or 9**^**th**^ **grade**	18	6.29
** 10**^**th**^ **grade**	71	24.83
** High school**	105	36.71
** University**	92	32.17
**Marital status**		
** Married**	105	36.71
** Never married**	171	59.79
** Separated/ widowed/ divorced**	10	3.50
**Working time**		
** Full-time job**	112	39.16
** Part-time job**	79	27.62
** Currently not working**	95	33.22
**Monthly household income (after taxes)**		
** Less than 500 Euros**	20	6.99
** 500–1,000 Euros**	98	34.27
** 1,500–2,500 Euros**	90	31.47
** 2,500–3,500 Euros**	53	18.53
** 3,500–4,500 Euros**	18	6.29
** More than 4,500 Euros**	7	2.45
**Birth characteristics**	N	%
**Parity**		
** Primiparous**	168	58.74
** Multiparous**	118	41.26
**Preterm delivery (N = 284)**		
** No**	274	96.48
** Yes (< 37 + 0 weeks of gestation)**	10	3.52
**Mode of delivery (N = 284)**		
** Spontaneous**	224	78.87
** Elective/ planned cesarean section**	19	6.69
** Emergency/ other cesarean section**	26	94.72
** Instrumental vaginal delivery**	15	5.28
**Anesthesia (N = 278)** ^2^		
** No/ other**	204	73.38
** Epidural anesthesia**	64	23.02
** General anesthesia**	10	3.60
**FOC/ subjective birth experience**		
** FOC (W-DEQ-A; N = 276)**	84.05	9.23
** Subjective birth experience (W-DEQ-B; N = 282)**	88.23	10.71
** Difference (W-DEQ-B minus W-DEQ-A; N = 272)**	4.13	9.92
**Big Five personality traits (M, SD)**		
** Openness**	3.78	0.63
** Conscientiousness**	3.84	0.58
** Extraversion**	3.59	0.83
** Agreeableness**	3.35	0.67
** Emotional stability**	3.35	0.80

Note: M = mean. SD = standard deviation.

^1^ All women of the MARI study who provided information on FOC (W-DEQ-A, T3, N = 276) and/ or subjective birth experiences (W-DEQ-B, T4, N = 282) were considered (N = 286). Specifically, 272 women provided information on the W-DEQ-A and W-DEQ-B, 4 women provided information on the W-DEQ-A but not W-DEQ-B, and 10 women provided information on the W-DEQ-B but not W-DEQ-A. For some birth characteristics, the N is slightly lower due to individual missing values.

^2^Of the women with an epidural anesthesia (N = 64), 27 women had a spontaneous delivery, 17 an elective/ planned cesarean section, 17 an emergency/ other cesarean section, and 3 an instrumental vaginal delivery.

### Associations of personality with FOC and subjective birth experiences

As shown in Table 2, lower emotional stability (*β* = -0.17), lower agreeableness (*β* = -0.16), and lower extraversion (*β* = -0.14) predicted higher FOC (crude models). However, only lower emotional stability (*β* = -0.13) remained a significant predictor of higher FOC when considering all Big Five personality traits simultaneously as multiple predictors (multiple model).

**Table 2:** Associations of personality with FOC (W-DEQ-A), subjective birth experiences (W-DEQ-B), and the discrepancy between subjective birth experiences and previous FOC (W-DEQ-B minus W-DEQ-A).

	FOC				Subjective birth experience				Difference between FOC and subjective birth experience			
	(W-DEQ-A)				(W-DEQ-B)							
									(W-DEQ-B minus W-DEQ-A)			
	N = 276				N = 282				N = 272			
Personality trait	β	95% CI		p	β	95% CI		p	β	95% CI		p
**Crude models**												
** Openness**	-0.03	-0.14	0.09	.661	-0.08	-0.19	0.04	.188	-0.07	-0.19	0.05	.257
** Conscientiousness**	-0.03	-0.14	0.09	.666	0.01	-0.11	0.13	.872	0.00	-0.12	0.13	.944
** Extraversion**	-0.14	-0.25	-0.02	.020	-0.06	-0.17	0.06	.316	0.07	-0.05	0.19	.280
** Agreeableness**	-0.16	-0.28	-0.05	.006	-0.02	-0.13	0.10	.763	0.12	0.00	0.25	.043
** Emotional stability**	-0.17	-0.28	-0.06	.004	0.01	-0.10	0.12	.863	0.16	0.04	0.27	.009
**Multiple models**												
** Openness**	0.01	-0.11	0.13	.836	-0.07	-0.19	0.05	.251	-0.10	-0.22	0.03	.119
** Conscientiousness**	0.06	-0.07	0.18	.366	0.03	-0.10	0.15	.663	-0.05	-0.18	0.08	.415
** Extraversion**	-0.08	-0.21	0.05	.212	-0.05	-0.18	0.07	.404	0.03	-0.10	0.16	.659
** Agreeableness**	-0.12	-0.24	0.00	.057	-0.01	-0.14	0.11	.834	0.10	-0.03	0.23	.139
** Emotional stability**	-0.13	-0.25	0.00	.045	0.03	-0.09	0.16	.609	0.15	0.02	0.28	.024

Note: β = standardized beta-coefficients from linear regressions, adjusted for age at baseline. CI = confidence interval.

Subjective birth experiences did not vary by personality (all *p*-values >.05). However, higher emotional stability (*β* = 0.16) and higher agreeableness (*β* = 0.12) predicted a greater discrepancy between subjective birth experiences and previous FOC (crude models). Thus, especially more emotionally stable and more agreeable women experienced their delivery as worse than expected. However, only emotional stability (*β* = 0.15) remained a significant predictor of this discrepancy when considering all Big Five personality traits simultaneously as multiple predictors (multiple model).

### The role of birth characteristics

Means and standard deviations for subjective birth experiences by birth characteristics are presented in S1 Table. Associations between birth characteristics and subjective birth experiences are shown in Table 3. Interactions between personality and birth characteristics in predicting subjective birth experiences are shown in S2 Table.

**Table 3:** Associations of birth characteristics with subjective birth experiences (W-DEQ-B) and the discrepancy between subjective birth experiences and previous FOC (W-DEQ-B minus W-DEQ-A).

	Subjective birth experience				Difference between FOC and subjective birth experience			
	(W-DEQ-B)							
					(W-DEQ-B minus W-DEQ-A)			
	N = 282				N = 272			
Birth characteristic	β	95% CI		p	β	95% CI		p
**Parity (1 vs. 0)**	-0.25	-0.50	0.00	.052	-0.44	-0.69	-0.18	.001
**Preterm delivery (1 vs. 0)**	0.25	-0.40	0.90	.455	-0.48	-1.30	0.33	.243
**Mode of delivery (1 vs. 0)**	0.31	-0.14	0.76	.178	0.31	-0.18	0.80	.218
**Mode of delivery (2 vs. 0)**	0.87	0.48	1.26	< .001	0.87	0.44	1.30	< .001
**Mode of delivery (3 vs. 0)**	0.37	-0.14	0.88	.158	0.38	-0.15	0.91	.157
**Anesthesia (1 vs. 0)**	0.67	0.41	0.94	< .001	0.33	0.04	0.61	.024
**Anesthesia (2 vs. 0)**	0.43	-0.18	1.03	.165	0.82	0.14	1.51	.019

Note: β = standardized beta-coefficients from linear regressions, adjusted for age at baseline. CI = confidence interval.

#### Parity

Subjective birth experiences did not differ between primiparous and multiparous women per se (all *p*-values >.05). However, the discrepancy between subjective birth experiences and previous FOC was smaller in multiparous vs. primiparous women (*β* = -0.44). That is, both primiparous and multiparous women experienced their delivery as worse than expected, but this discrepancy was smaller in multiparous (vs. primiparous) women.

Moreover, the role of emotional stability in the discrepancy between subjective birth experiences and previous FOC differed between primiparous and multiparous women (*β* = 0.27, 95% CI: 0.03; 0.51, *p* = .029): Only in multiparous women (*β* = 0.31, 95% CI: 0.12; 0.50, *p* = .002) but not in primiparous women (*p*-value >.05), higher emotional stability predicted a greater discrepancy between subjective birth experiences and previous FOC. In other words, especially multiparous women with high levels of emotional stability tended to experience their delivery as worse than expected.

#### Preterm delivery

Subjective birth experiences did not differ between women with and without a preterm delivery (all *p*-values >.05). However, the role of emotional stability in subjective birth experiences differed between women with and without a preterm delivery (*β* = -1.11, 95% CI: -1.78; -0.44, *p* = .001): Only in women with a preterm delivery (*β* = -1.24, 95% CI: -2.12; -0.35, *p* = .014) but not in women without a preterm delivery (*p*-value >.05), lower emotional stability predicted worse subjective birth experiences.

#### Mode of delivery

As expected, women with an emergency cesarean section experienced their delivery as worse than women with a spontaneous delivery (*β* = 0.87). Consistently, the discrepancy between subjective birth experiences and previous FOC was greater in women with an emergency cesarean section compared to women with a spontaneous delivery (*β* = 0.87). That is, both women with a spontaneous delivery and an emergency cesarean section experienced their delivery as worse than expected, but this discrepancy was greater in women with an emergency cesarean section (vs. spontaneous delivery).

Furthermore, the role of conscientiousness in subjective birth experiences differed between women with a spontaneous delivery and an emergency cesarean section (*β* = 0.53, 95% CI: 0.14; 0.92, *p* = .008): Only in women with an emergency cesarean section (*β* = 0.46, 95% CI: 0.01; 0.91, *p* = .047) but not in women with a spontaneous delivery (*p*-value >.05), higher conscientiousness predicted worse subjective birth experiences.

#### Anesthesia during delivery

Compared to women without anesthesia during delivery, women with epidural anesthesia reported higher FOC (*β* = 0.36) and experienced their delivery as worse (*β* = 0.67). Moreover, the discrepancy between subjective birth experiences and previous FOC was greater in women with epidural anesthesia (*β* = 0.33) and general anesthesia (*β* = 0.82) compared to women without anesthesia during delivery. That is, especially women who received anesthetics experienced their delivery as worse than expected.

In addition, the role of openness in subjective birth experiences differed between women without anesthesia and women with general anesthesia (*β* = 1.14, 95% CI: 0.59; 1.69, *p* < .001): Only in women without anesthesia (*β* = -0.15, 95% CI: -0.29; -0.02, *p* = .023) but not in women with general anesthesia (*p*-value >.05), lower openness predicted worse subjective birth experiences.

Similarly, the role of conscientiousness in subjective birth experiences differed between women without anesthesia and women with general anesthesia (*β* = 1.79, 95% CI: 1.01; 2.57, *p* < .001): Only in women with general anesthesia (*β* = 1.73, 95% CI: 0.60; 2.87, *p* = .009) but not in women without anesthesia (*p*-value >.05), higher conscientiousness predicted worse subjective birth experiences.

Finally, the role of emotional stability in the discrepancy between subjective birth experiences and previous FOC differed between women without anesthesia and women with epidural anesthesia during delivery (*β* = -0.28, 95% CI: -0.55; -0.01, *p* = .041): Only in women without anesthesia (*β* = 0.23, 95% CI: 0.10; 0.36, *p* < .001) but not in women with epidural anesthesia (*p*-value >.05), higher emotional stability predicted a greater discrepancy between subjective birth experiences and previous FOC. In other words, especially highly emotional stable women without anesthesia experienced their delivery as worse than expected.

## Discussion

This prospective-longitudinal study examined the role of expectant mothers’ personality in FOC and subjective birth experiences. Our main findings are as follows: First, less emotionally stable, less agreeable, and less extraverted women experienced higher FOC. Second, only in women with specific birth characteristics—but not in the total sample—subjective birth experience varied as a function of women’s personality. Third, especially more emotionally stable women experienced their delivery as worse than expected. In particular, this applied to multiparous women and women without anesthesia during delivery.

### Associations between personality and FOC

Consistent with previous research [30–33, 35], we found that especially less emotionally stable women, but also less agreeable and less extraverted women experienced higher FOC. Less emotionally stable individuals are more prone to anxiety [38], which might explain a higher susceptibility to FOC [30–33, 35]. Moreover, less agreeable and less extraverted women might have fewer and less supportive social relationships and thus receive lower levels of social support in order to cope with birth-related fears [13, 43].

### Associations between personality and subjective birth experiences

Only in women with specific birth characteristics—but not in the total sample—subjective birth experiences varied as a function of women’s personality. Specifically, in women with (but not without) a preterm delivery, lower emotional stability predicted worse subjective birth experiences. This finding is consistent with our hypothesis that personality would affect subjective birth experiences more strongly in women with (vs. without) a preterm delivery. A preterm delivery often sets in unexpectedly and relates to a higher risk of unfavorable outcomes. Especially less emotionally stable women might perceive this frequently unexpected and challenging situation as threatening, which could explain our results.

Our study further revealed that in women with an emergency cesarean section (but not spontaneous delivery), higher conscientiousness was associated with worse subjective birth experiences. Similarly, in women with (but not without) general anesthesia, higher conscientiousness predicted worse subjective birth experiences. Highly conscientious individuals are characterized by high levels of mastery and self-control [38, 39]. An emergency cesarean section, however, means being unable to manage the birth “independently” and relates to a loss of control—especially under general anesthesia. For highly conscientious women, this experience might be particularly adverse.

Finally, only in women without (but not with) anesthesia during delivery, lower openness was associated with worse subjective birth experiences. Particularly women without anesthesia have to manage a variety of bodily perceptions during delivery. For less open-minded women, this challenge might be more difficult.

### Associations between personality and the discrepancy between subjective birth experiences and previous FOC

Furthermore, especially more emotionally stable women experienced their delivery as worse than expected. In particular, this applied to multiparous women and women without anesthesia during delivery. In our study, more emotionally stable women reported lower FOC, but emotional stability was unrelated to women’s subjective birth experiences. Taken together, this might suggest that highly emotionally stable women tend to underestimate the burden and pain of the upcoming birth, particularly if they feel used to it due to previous deliveries and/ or receive no anesthetics. Our findings are particularly noteworthy given the fact that the discrepancy between women’s birth expectancies and experiences has received little attention so far.

### Strengths and limitations

Our study has various strengths: Our data come from a prospective-longitudinal study. Personality at the beginning of pregnancy (BFI-K), FOC shortly before delivery (W-DEQ-A), and subjective birth experiences shortly after delivery (W-DEQ-B) were assessed using well-established questionnaires with solid psychometric properties. As one of the first studies, we not only considered FOC and subjective birth experiences but also the discrepancy between subjective birth experiences and previous FOC. Moreover, we tested whether the role of personality in subjective birth experiences varies by birth characteristics.

Though, our study is not without limitations: First, although subjective birth experiences were assessed shortly after delivery, these self-reports might have been affected by retrospective recall and memory biases. For example, some participants might have under-rated negative experiences during labor and delivery due to the joy about their child afterwards. However, the W-DEQ-B was applied during the first 10 days after delivery and has excellent psychometric properties [51–53]. Capturing subjective birth experiences in time to experience (i.e., during delivery) would hardly be possible.

Second, a few women did not participate in single assessments, for example, due to a preterm delivery, sickness, or lack of time, and we cannot rule out the possibility that these missing values occurred systematically. However, the retention rate in the MARI study was excellent [48, 49], and 286 (93.5%) women out of the initial total sample (*N* = 306) could be considered for the current analyses. Third, our findings stem from a regional-epidemiological sample from Dresden in Germany and might not be generalizable to pregnant women in general [48, 49].

## Conclusions

Our study suggests that women’s personality may affect both their birth expectancies and birth experiences: Especially less emotionally stable women with a preterm delivery, more conscientious women with an emergency cesarean section (under general anesthesia), and less open women without anesthesia during delivery tend to experience their delivery as worse. Moreover, especially more emotionally stable multiparous women seem to anticipate less adverse birth experiences, which might result in subjective birth experiences that are worse than expected. Future research is warranted to replicate our findings and to examine the efficacy of targeted interventions to prevent a vicious cycle of severe FOC, adverse subjective birth experiences, and associated unfavorable outcomes in expectant mothers and their offspring [25–27]. Such research may also investigate the effects on further family planning, subsequent pregnancies, and future birth experiences.

## Supporting information

S1 Table Means and standard deviations for FOC (W-DEQ-A), subjective birth experiences (W-DEQ-B), and the discrepancy between subjective birth experiences and previous FOC (W-DEQ-B minus W-DEQ-A) by birth characteristics. (DOCX)

S2 Table Interactions between personality and birth characteristics in predicting subjective birth experiences (W-DEQ-B), and the discrepancy between subjective birth experiences and previous FOC (W-DEQ-B minus W-DEQ-A). (DOCX)
