# Supplementary material for: Personality impacts fear of childbirth and subjective birth experiences: A prospective-longitudinal study
Source: PLoS One. 2021 Nov 3;16(11):e0258696. doi: 10.1371/journal.pone.0258696 (PMC8565718; doi:10.1371/journal.pone.0258696)
Supplement: S1 Table — (DOCX) [file pone.0258696.s001.docx]

**S1 Table. Means and standard deviations for FOC (W-DEQ-A), subjective birth experiences (W-DEQ-B), and the discrepancy between subjective birth experiences and previous FOC (W-DEQ-B minus W-DEQ-A) by birth characteristics**

|  | **Subjective birth experience**  **(W-DEQ-B)** | | **Difference between FOC and subjective birth experience**  **(W-DEQ-B minus W-DEQ-A)** | |
| --- | --- | --- | --- | --- |
|  | **N = 282** | | **N = 272** | |
| **Birth characteristic** | **M** | **SD** | **M** | **SD** |
| **Parity** |  |  |  |  |
| **(0) Primiparous** | 88.39 | 10.59 | 5.54 | 9.85 |
| **(1) Multiparous** | 88.00 | 10.91 | 2.08 | 9.72 |
| **Preterm delivery** |  |  |  |  |
| **(0) No** | 88.12 | 10.54 | 4.23 | 9.91 |
| **(1) Yes (< 37 + 0 weeks of gestation)** | 91.43 | 15.48 | -0.53 | 10.58 |
| **Mode of delivery** |  |  |  |  |
| **(0) Spontaneous** | 86.96 | 9.76 | 3.04 | 9.71 |
| **(1) Elective/planned cesarean section** | 91.96 | 14.55 | 6.38 | 10.79 |
| **(2) Emergency/other cesarean section** | 95.64 | 13.46 | 11.59 | 9.51 |
| **(3) Instrumental vaginal delivery** | 90.14 | 7.58 | 6.71 | 7.28 |
| **Anesthesia** |  |  |  |  |
| **(0) No/ other** | 86.47 | 9.65 | 3.25 | 9.14 |
| **(1) Epidural anesthesia** | 93.67 | 10.96 | 6.48 | 11.39 |
| **(2) General anesthesia during cesarean section** | 90.38 | 18.59 | 11.38 | 3.47 |

Note: M = mean. SD = standard deviation.
