# Supplementary material for: Personality impacts fear of childbirth and subjective birth experiences: A prospective-longitudinal study
Source: PLoS One. 2021 Nov 3;16(11):e0258696. doi: 10.1371/journal.pone.0258696 (PMC8565718; doi:10.1371/journal.pone.0258696)
Supplement: S2 Table — (DOCX) [file pone.0258696.s002.docx]

**S2 Table. Interactions between personality and birth characteristics in predicting subjective birth experiences (W-DEQ-B), and the discrepancy between subjective birth experiences and previous FOC (W-DEQ-B minus W-DEQ-A)**

|  | **Subjective birth experience**  **(W-DEQ-B)** | | | | **Difference between FOC and subjective birth experience**  **(W-DEQ-B minus W-DEQ-A)** | | | |
| --- | --- | --- | --- | --- | --- | --- | --- | --- |
|  | **N = 282** | | | | **N = 272** | | | |
| **Interaction term** | **β** | **95% CI** | | **p** | **β** | **95% CI** | | **p** |
| **Parity (1 vs. 0)** |  |  |  |  |  |  |  |  |
| **Openness** | -0.02 | -0.25 | 0.21 | .839 | -0.09 | -0.33 | 0.15 | .443 |
| **Conscientiousness** | 0.17 | -0.07 | 0.41 | .155 | 0.16 | -0.08 | 0.41 | .197 |
| **Extraversion** | -0.04 | -0.27 | 0.19 | .732 | -0.04 | -0.28 | 0.20 | .748 |
| **Agreeableness** | -0.02 | -0.25 | 0.22 | .898 | 0.04 | -0.21 | 0.29 | .760 |
| **Emotional stability** | 0.16 | -0.08 | 0.39 | .201 | 0.27 | 0.03 | 0.51 | .029 |
| **Preterm delivery (1 vs. 0)** |  |  |  |  |  |  |  |  |
| **Openness** | -0.72 | -1.59 | 0.15 | .103 | -0.95 | -2.27 | 0.38 | .159 |
| **Conscientiousness** | 0.19 | -0.74 | 1.11 | .690 | 0.20 | -0.77 | 1.18 | .681 |
| **Extraversion** | -0.53 | -1.08 | 0.02 | .058 | -0.61 | -1.29 | 0.06 | .075 |
| **Agreeableness** | 0.00 | -0.81 | 0.81 | .997 | -0.13 | -1.02 | 0.76 | .772 |
| **Emotional stability** | -1.11 | -1.78 | -0.44 | .001 | -0.63 | -1.38 | 0.13 | .105 |
| **Mode of delivery (1 vs. 0)** |  |  |  |  |  |  |  |  |
| **Openness** | 0.18 | -0.21 | 0.56 | .361 | -0.20 | -0.62 | 0.22 | .346 |
| **Conscientiousness** | 0.46 | -0.02 | 0.94 | .058 | 0.25 | -0.31 | 0.81 | .383 |
| **Extraversion** | 0.54 | -0.09 | 1.17 | .091 | 0.04 | -0.62 | 0.70 | .905 |
| **Agreeableness** | 0.39 | -0.08 | 0.86 | .106 | 0.03 | -0.52 | 0.58 | .921 |
| **Emotional stability** | 0.34 | -0.24 | 0.91 | .251 | -0.15 | -0.75 | 0.45 | .614 |
| **Mode of delivery (2 vs. 0)** |  |  |  |  |  |  |  |  |
| **Openness** | 0.07 | -0.31 | 0.44 | .720 | 0.16 | -0.26 | 0.57 | .461 |
| **Conscientiousness** | 0.53 | 0.14 | 0.92 | .008 | 0.26 | -0.15 | 0.67 | .218 |
| **Extraversion** | -0.07 | -0.52 | 0.37 | .741 | -0.19 | -0.71 | 0.33 | .480 |
| **Agreeableness** | 0.06 | -0.30 | 0.42 | .741 | 0.00 | -0.38 | 0.39 | .982 |
| **Emotional stability** | 0.06 | -0.28 | 0.40 | .728 | -0.03 | -0.38 | 0.33 | .883 |
| **Mode of delivery (3 vs. 0)** |  |  |  |  |  |  |  |  |
| **Openness** | -0.16 | -0.70 | 0.38 | .561 | -0.21 | -0.76 | 0.35 | .465 |
| **Conscientiousness** | -0.33 | -0.82 | 0.15 | .175 | -0.09 | -0.60 | 0.42 | .719 |
| **Extraversion** | -0.09 | -0.68 | 0.50 | .758 | -0.37 | -0.98 | 0.24 | .233 |
| **Agreeableness** | -0.33 | -0.82 | 0.15 | .178 | -0.26 | -0.76 | 0.25 | .315 |
| **Emotional stability** | -0.19 | -0.78 | 0.41 | .542 | -0.56 | -1.17 | 0.04 | .068 |
| **Anesthesia (1 vs. 0)** |  |  |  |  |  |  |  |  |
| **Openness** | 0.18 | -0.06 | 0.41 | .147 | 0.08 | -0.17 | 0.34 | .524 |
| **Conscientiousness** | 0.08 | -0.19 | 0.34 | .566 | 0.01 | -0.27 | 0.29 | .964 |
| **Extraversion** | -0.03 | -0.33 | 0.26 | .813 | -0.12 | -0.44 | 0.19 | .449 |
| **Agreeableness** | 0.18 | -0.08 | 0.44 | .181 | 0.14 | -0.14 | 0.42 | .334 |
| **Emotional stability** | -0.19 | -0.46 | 0.07 | .148 | -0.28 | -0.55 | -0.01 | .041 |
| **Anesthesia (2 vs. 0)** |  |  |  |  |  |  |  |  |
| **Openness** | 1.14 | 0.59 | 1.69 | <.001 | 0.09 | -1.10 | 1.28 | .888 |
| **Conscientiousness** | 1.79 | 1.01 | 2.57 | <.001 | 0.31 | -0.75 | 1.38 | .563 |
| **Extraversion** | 0.80 | -0.32 | 1.92 | .160 | -0.22 | -1.64 | 1.21 | .765 |
| **Agreeableness** | 0.36 | -0.17 | 0.89 | .182 | -0.05 | -0.71 | 0.60 | .876 |
| **Emotional stability** | -0.08 | -0.97 | 0.81 | .855 | -0.44 | -1.40 | 0.51 | .362 |

Note: β = standardized beta-coefficients from linear regressions, adjusted for age at baseline. CI = confidence interval.
